# mirCoX: a database of miRNA-mRNA expression correlations derived from RNA-seq meta-analysis

**DOI:** 10.1186/1471-2105-14-S14-S17

**Published:** 2013-10-09

**Authors:** Cory B Giles, Reshmi Girija-Devi, Mikhail G Dozmorov, Jonathan D Wren

**Affiliations:** 1 Oklahoma Medical Research Foundation, Oklahoma City, Arthritis and Clinical Immunology Research Program, 825 N.E. st, Oklahoma City, OK 73104-5005, USA; 2 University of Oklahoma Health Sciences Center, Department of Biochemistry and Molecular Biology, 940 Stanton L. Young Blvd, OK 73104-5005, USA; 3 Rajiv Gandhi Centre for Biotechnology, Thiruvananthapuram, Kerala, India

## Abstract

**Background:**

Experimentally validated co-expression correlations between miRNAs and genes are a valuable resource to corroborate observations about miRNA/mRNA changes after experimental perturbations, as well as compare miRNA target predictions with empirical observations. For example, when a given miRNA is transcribed, true targets of that miRNA should tend to have lower expression levels relative to when the miRNA is not expressed.

**Methods:**

We processed publicly available human RNA-seq experiments obtained from NCBI's Sequence Read Archive (SRA) to identify miRNA-mRNA co-expression trends and summarized them in terms of their Pearson's Correlation Coefficient (PCC) and significance.

**Results:**

We found that sequence-derived parameters from TargetScan and miRanda were predictive of co-expression, and that TargetScan- and miRanda-derived gene-miRNA pairs tend to have anti-correlated expression patterns in RNA-seq data compared to controls. We provide this data for download and as a web application available at http://wrenlab.org/mirCoX/.

**Conclusion:**

This database of empirically established miRNA-mRNA transcriptional correlations will help to corroborate experimental observations and could be used to help refine and validate miRNA target predictions.

## Background

MicroRNAs (miRNAs) are small (19-22 nt) non-coding RNAs that can interfere with mRNA translation by base-pairing with mRNAs to form double-stranded RNA or by promoting the loss of polyadenylation, leading to inhibition of translation or degradation of the mRNA by the cellular machinery [1,2]. Less often, dsRNA formed by miRNA-target complexes can target gene promoters and actually enhance transcription of target genes, sometimes termed RNAa (RNA activation) [3]. Through these mechanisms, a single miRNA can potentially alter the expression levels of hundreds, even thousands, of mRNA transcripts [4] and long non-coding RNAs [5] and therefore exert considerable regulatory control over cellular processes. The diverse regulatory behavior of microRNAs also has been found to play an important role in a wide variety of pathologies, including cancer and cardiovascular disease [6,7].

Predicting miRNA target genes has been a topic of active research [8], and works by calculating sequence similarity metrics between miRNAs and their putative target sequences, often in the 3' UTR of genes [9-14], although other features have predictive value [15]. However, because miRNA-target base pairing is rarely characterized by perfect complementarity, even in the "seed" region, sequence-based miRNA target prediction algorithms are prone to many false positives and have poor inter-algorithm agreement [16,17]. Experimental validation of miRNA-target interactions can be conducted, but is prohibitively costly and time-consuming to do for large-scale assessments of miRNA target prediction efficacy. Because of the biological effects of miRNAs on their targets, expression data is something that can potentially assist sequence-based methods. Therefore, several methods have been developed to prioritize predicted miRNA-target interactions *in silico*.

Several groups [18-20] have used expression data from paired miRNA-mRNA microarrays to generate correlations between miRNAs and their putative targets, with the idea that miRNA-mRNA pairs with a negative correlation are more likely to be legitimate interactions [21]. Similarly, Gennarino *et al *developed HOCTAR, a method that re-ranks sequence-based predictions for intragenic miRNAs by using expression of a host gene as a proxy for expression of the miRNA, thus avoiding the necessity of using specialized miRNA arrays but at the cost of being restricted to intragenic miRNAs [21,22].

Although previous expression-based miRNA-target interaction prioritization approaches have been successful, they have several drawbacks. Typically, the miRNA and mRNA expression profiles are determined by different types of arrays, leading to the possibility of technology-specific artifacts or batch effects. Although microRNAs can target other non-coding RNAs and methods are being actively developed to discover such interactions [5,23,24], ncRNAs are poorly represented on most array platforms. Furthermore, microarrays are not as quantitative and require probes for each transcript of interest, as compared with next-generation sequencing technology, which is probe agnostic and can provide information about all expressed transcripts, including isoforms.

To overcome these drawbacks, we generated a database of expression correlations between microRNAs and mRNAs by using total RNA sequencing (RNA-seq) experiments from NCBI's Sequence Read Archive (SRA). We then integrated sequence-based miRNA target predictions from miRanda and TargetScan databases together with RNA-seq derived expression correlations to prioritize these predicted miRNA-target pairs by co-expression and created a publicly-available web server for users to query these expression correlations. We anticipate this will be potentially useful for two different types of users. First, biologists would be interested from the standpoint of interpreting correlations in their own experiments. For example, if they knocked out Gene X and observed miRNA Y was highly expressed in that experiment, a natural question to ask is whether or not X and Y are normally anti-correlated. If so, it lends itself to the hypothesis that Gene X might somehow repress miRNA Y, either directly or indirectly. Second, bioinformaticists would potentially be interested in downloading the data to either help train their sequence-based miRNA target prediction algorithms or to use the correlations as corroborating evidence of effect. Finally, we would like to note that, although the empirical correlations we are reporting here may be suggestive of effect, this resource itself is not intended to predict miRNA-mRNA target pairs.

## Methods

### Selection and pre-processing of RNA-seq experiments

RNA co-expression data was obtained from processing RNA-seq datasets available for download in NCBI's Sequence Read Archive (SRA). The Bioconductor package SRAdb was installed [25] and its companion database was downloaded, current as of January 2013, to obtain experiment information from SRA. The database was queried to find RNA-seq runs which met the following criteria: 1) Taxon ID 9606 (human), 2) "RANDOM" library selection, to ensure that certain varieties of transcripts were not artificially enriched or depleted by the selection method, as for example via poly(A) tail selection; 3) "TRANSCRIPTOMIC" library sources and "RNA-Seq" library strategy to eliminate specialized procedures such as cap analysis of gene expression (CAGE), and 4) paired-end reads. The selected run accessions were then downloaded from the SRA using the Aspera software and converted to FASTQ using the "fastq-dump" program from NCBI's SRA Toolkit [26].

Reads were trimmed first for Illumina adapters, then by quality, using the "fastq-mcf" program from the ea-utils package. The Bowtie2 aligner [27] was used to map each set of reads in FASTQ format to the GRCh37/hg19 reference genome using the "--sensitive" parameter. Samtools [28] was used to convert Bowtie2's SAM output to sorted BAM. Using the Kent source utilities [29], these mappings were then converted to BigWig, and transcript coverage was quantified using the "bigWigAverageOverBed" program, where transcript coordinates were obtained from the UCSC knownGenes (for genes and ncRNAs) and from mirBase (for microRNAs). To obtain runs with adequate sequencing depth and quality, runs which had fewer than 10 million mapped reads, fewer than 60% mapped reads, or which failed two or more FASTQC tests were discarded from further analysis. Overall, two expression matrices (of knownGene IDs and mirBase IDs) containing raw mapped counts were constructed using 141 runs (see Additional File 1 for a list of runs used).

### Transcript-miRNA correlations and miRNA-target information

**Figure 1:**
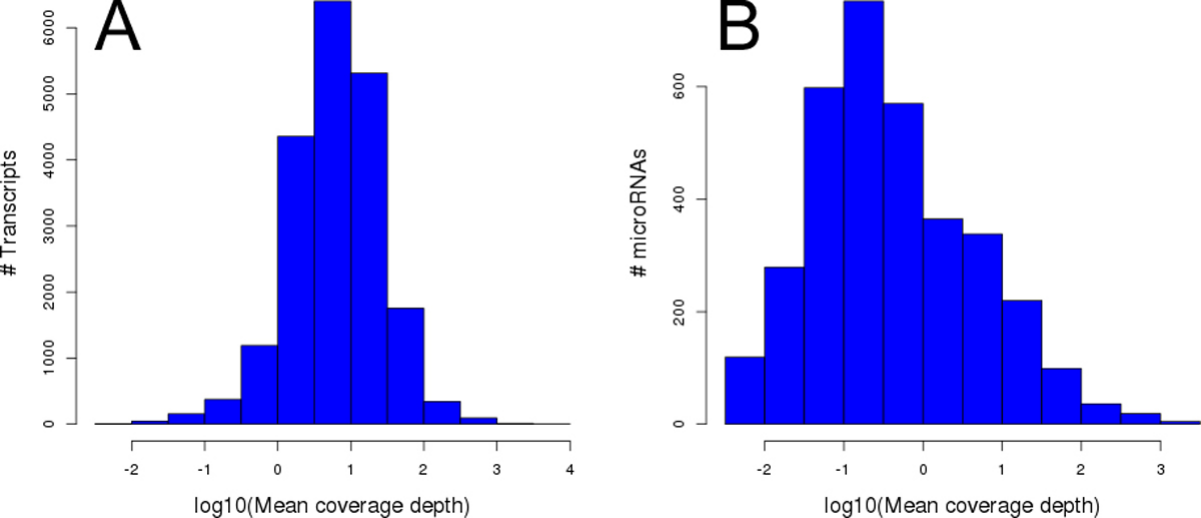
**Distribution of miRNAs and gene expression in RNA-seq samples**. For each sequencing run, the read depth is calculated using the "bigWigAverageOverBed" utility. The per-transcript read depth averaged across all sequencing runs for coding and non-coding transcripts (a) and microRNAs (b) shows a typical log-normal distribution.

Both expression matrices (of knownGene IDs and mirBase IDs) were normalized using the implementation of quantile normalization provided by the limma package [30]. As shown in Figure 1, both genes and miRs showed a typical log-normal distribution of average mapped read depth, although it is likely that many of the miRs detected were in their precursor (pre-miRNA) forms. Pearson correlation coefficients and quantile expression rank between transcripts (knownGene IDs) and miRNAs were obtained using these two matrices and the Numpy suite of programs. Transcripts or miRNAs which were not detected in any experiment were not assigned correlations and were removed from downstream analyses. In total, 58282 of the UCSC knownGenes and 597 miRNAs were detected in at least one experiment.

Experimentally validated target predictions for each miRNA were obtained from miRecord [31], and computational predictions were obtained from TargetScan version 6.2 [12] and miRanda (August 2010 release) [9,11]. miRanda divides its predicted targets into conserved and nonconserved targets, and we analyzed these two groups separately. For miRecord, only 215 of 1582 miR-target pairs were mappable to miR-knownGene pairs. The reason for this relatively low mapping rate is that frequently the RefSeq IDs for genes in miRecords did not correspond to a RefSeq ID for a knownGene transcript.

### Web application implementation

The above data were assembled into a MySQL database, and a PHP front-end was implemented to serve user searches on particular transcripts, genes, or miRNAs. The application is hosted on a LAMP server. This web interface is accessible by Internet Explorer version 10 or greater and modern versions of Firefox and Chrome.

## Results

To test the hypothesis that RNA-seq derived miRNA-mRNA transcriptional correlations could be used as a different data type to corroborate sequence-based methods, we examined the transcriptional correlations between predicted or experimentally validated miR-target pairs. Because the general mode of action for microRNAs is to repress their targets, valid miR-target pairs should have lower Pearson correlations than randomly selected miR-target pairs. Table 1 shows that this is the case for experimentally validated miR-target pairs (from miRecord) as well as for computationally predicted pairs (TargetScan, miRanda). The effect is most pronounced in the experimentally validated pairs, suggesting that experimentally validated pairs have the highest quality.

**Table 1:** miR-target correlations in experimentally validated and computationally predicted subsets

Dataset	Experimentally validated?	# miR-gene pairs	ΔCorrelation
miRecord	yes	268	-0.051
miRanda (conserved)	no	194414	-0.009
miRanda(nonconserved)	no	965520	-0.016
TargetScan	no	51487	-0.015

For each miR-target database, the mean miR-gene expression correlation was calculated for miR-target pairs within the database and compared to the mean correlation for all possible miR-gene pairs. **The difference between the mean correlation for pairs within a database and the mean correlation for all pairs is given as ∆correlation.**

The TargetScan and miRanda software provide several metrics that are used to predict the likelihood of a putative miRNA-mRNA interaction based on sequence and genomic location. To determine whether these scoring metrics were predictive of miRNA-mRNA pair expression correlations in our data, we determined the correlation between each parameter and the co-expression value for the corresponding miR-mRNA pair. For miRanda conserved and non-conserved targets, all parameters (alignment score, conservation score, free energy, and mirSVR score) were able to significantly predict co-expression values (Tables 2a, 2b). For TargetScan, all metrics except the number of non-conserved 8mer sites showed significant ability to predict co-expression values (Table 2c).

**Table 2:** miRanda and TargetScan score parameters can predict co-expression

Data Source	Parameter	Pearson Correlation	-log10 (P-Value)
miRanda conserved	Alignment Score	0.041	69.83
	Conservation Score	-0.023	22.16
	Free Energy	0.024	23.33
	mirSVR Score	-0.02	16.6
			
miRanda nonconserved	Alignment Score	0.026	139.55
	Conservation Score	-0.025	130.38
	Free Energy	-0.08	> 307
	mirSVR Score	0.024	120.49
			
TargetScan	Aggregate PCT	0.023	5.51
	Context Score	-0.112	141.13
	# Conserved Sites	0.0299	9.61
	# Nonconserved Sites	-0.064	45.92
	# Conserved 7mer-1a sites	0.019	3.27
	# Conserved 7mer-m8 sites	0.014	1.39
	# Conserved 8mer sites	0.02	4.2
	# Nonconserved 7mer-1a sites	-0.094	99.64
	# Nonconserved 7mer-m8 sites	0.031	10.64
	# Nonconserved 8mer sites	-0.008	0

For each predicted miRNA-mRNA pair from the miRanda conserved, miRanda nonconserved, and TargetScan datasets, the Pearson correlation coefficient for co-expression across multiple RNA-seq experiments was obtained. The correlation coefficient was then compared to various predictor variables provided by miRanda/TargetScan, across all pairs, again using Pearson correlation. (P-values after Bonferroni correction; * indicates significance at *p *< 0.05; ** indicates significance at *p *< 0.001).

**Figure 2:**
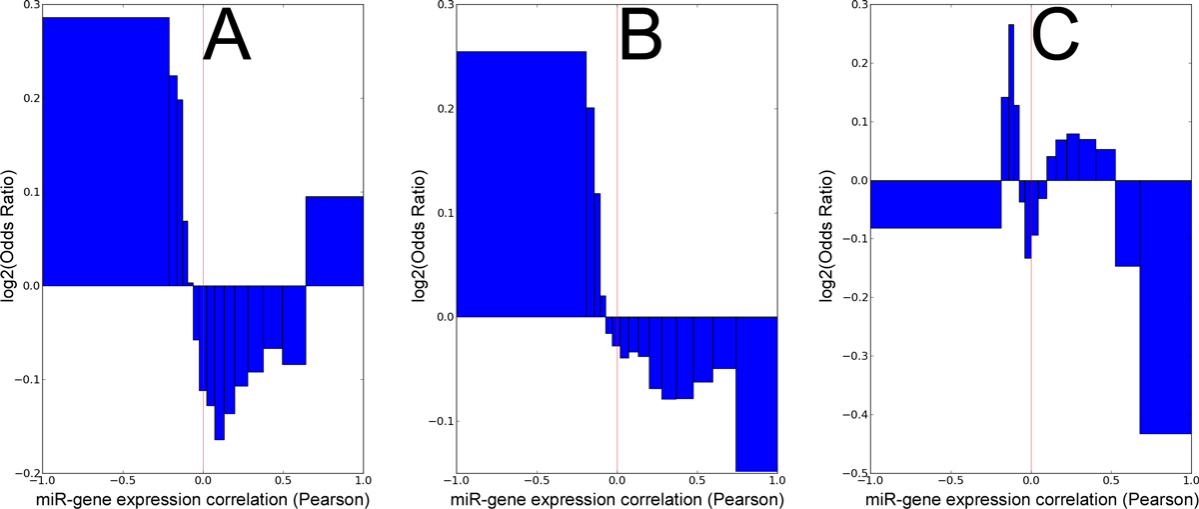
**Gene-miR pairs with negative expression correlations are overrepresented in predicted target databases**. Pearson correlations were calculated for the subset of gene-miR pairs found in the miRanda and TargetScan databases, and binned by correlation value. For each bin, the ratio of observed to expected pairs (odds ratio) was calculated. Bins which have a positive log odds ratio are over-represented in predicted targeting pairs, and bins with negative log odds ratio are under-represented. miR-gene pairs with negative expression correlation are clearly enriched in miRanda predicted interacting pairs, whereas TargetScan shows a bimodal enrichment pattern. A) miRanda conserved, B) miRanda nonconserved, C) TargetScan.

Next, we assessed the degree of agreement between mRNA-miRNA co-expression values and TargetScan predictions. Because miRNAs often repress expression of their targets, we hypothesized that gene-miR pairs with negative expression correlations should be enriched for target interactions predicted by miRanda and TargetScan. As expected, Figures 2a and 2b show a clear over-representation of miRanda conserved and non-conserved predictions among pairs with negative expression correlations. Figure 2c shows a more complex bimodal pattern, wherein TargetScan miR-target pairs tend to have moderately negative correlations, and to a lesser extent moderately positive correlations, but tend not to have extreme positive or negative correlations, or to be uncorrelated.

### Web application

**Figure 3:**
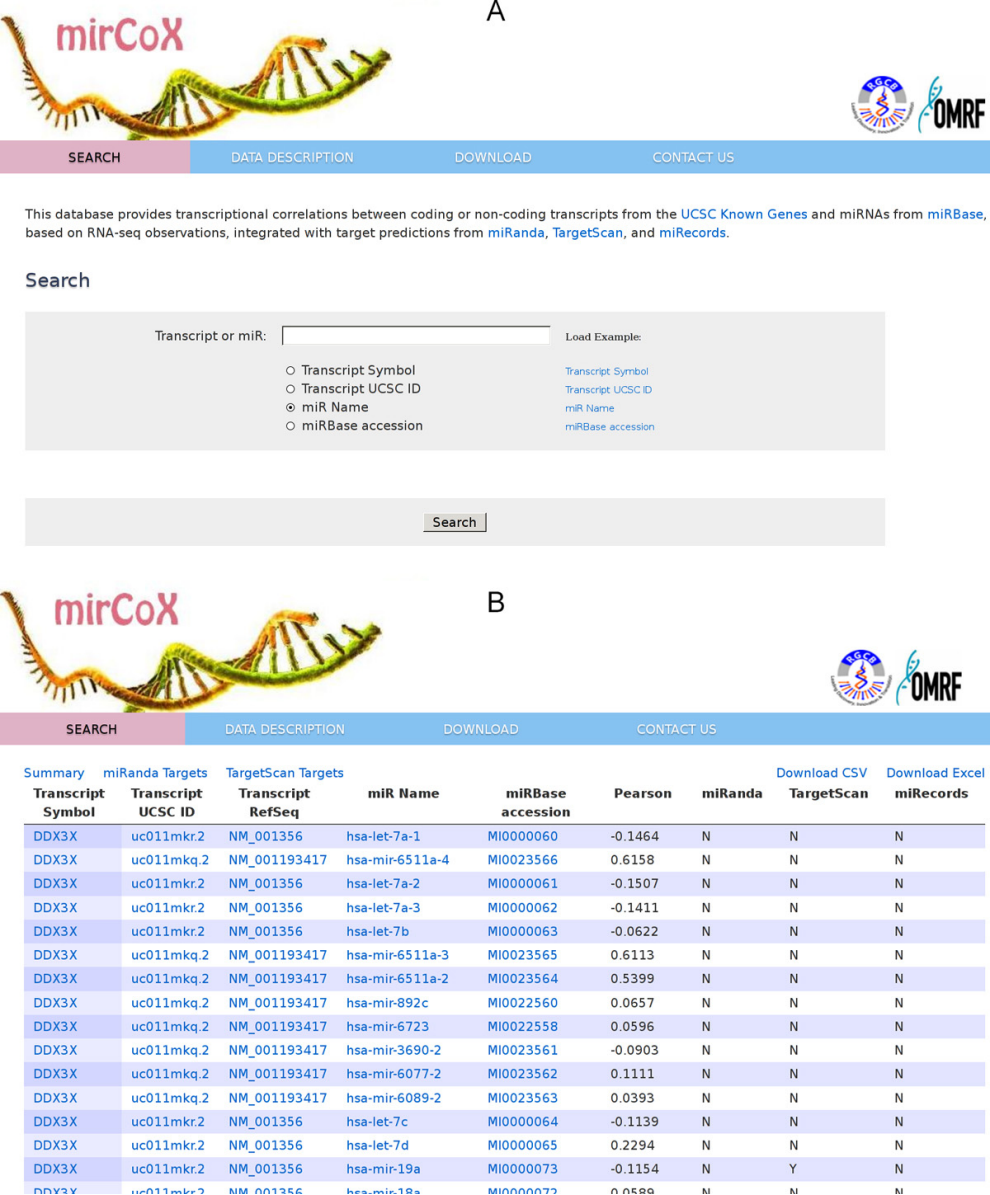
**mirCoX web server user interface**. (A) Screen capture of mirCoX application showing the online search screen. (B) A list of co-relational microRNA datasets based on the user's query by gene symbol and a menu bar with operations which can be performed on the data.

We created a web application (Figure 3) and tab-delimited source data files available for download. Gene/ncRNA or miRNA accessions can be entered to obtain a ranked list of positive and negative correlations with other transcripts. If a given gene-miR pair is predicted to interact by TargetScan or miRanda, the corresponding parameters, such as context score and numbers of conserved and non-conserved binding sites of each type, are also displayed alongside the expression values. The results of any query can be exported to CSV format, and bulk download of all the data used in the web server is also available at the same URL.

## Discussion

RNA-sequencing data contains information about expression patterns for the entire transcriptome at the time of measurement. Consequently, this data is an excellent means of exploring expression correlations among non-coding transcripts, microRNAs, and genes. We provide a method and interface to explore detected miRNA-mRNA correlations in light of the commonly accepted hypothesis that miRNA-mRNA pairing affects expression and/or translation of the mRNA, often as a means of repression but also as a means of activating transcription. Positive or negative correlations alone, of course, do not prove causation, as there can be a number of different factors involved in mRNA degradation. But strong correlations, particularly in the absence of other strong miRNA correlations with the same mRNA, can be considered strongly suggestive of an influence. And the more expression samples gathered for analysis, the more statistical confidence in trends that can be gained, and rare transcripts will become less of a problem.

The correlations we detected were statistically significant, yet much smaller in magnitude than we had anticipated prior to the study. There are a number of potential reasons this might be. First, since it has been observed that miRNAs, to achieve effective translational repression, frequently act in combination [32]., the transcription of one miRNA simply does not correlate strongly enough. This is analogous to the situation whereby multiple transcription factors frequently act in combination in order to achieve transcriptional activation [33]. Second, it is possible that the repressive effect is more pronounced on the translational level. If so, this would suggest transcriptional approaches are not well-suited to address this problem. Third, it's possible that there is another layer of regulation not accounted for by simple correlations, for example the competing endogenous RNA hypothesis, whereby some transcripts may exist to "soak up" multiple miRNAs of the same type and keep them from repressing their targets [34]. Finally, although probably least likely, if the bulk of transcripts being detected are pre-miRNAs that have not yet been processed (which we would expect to correlate with mature miRNA levels), then it's possible that there's another regulatory layer that keeps them from being processed until some further signal is given.

In summary, RNA-seq experiments provide us with a unique overview of the whole transcriptome in a single experiment, which can be used to detect transcript-transcript correlations and potentially detect whether or not one type of transcript with known regulatory effects is influencing the other. This work provides a resource to examine predicted correlations among two classes of RNA that are known to interact, with miRNAs able to degrade mRNA expression. It has the potential to help corroborate sequence-based predictions of miRNA-mRNA interactions, estimate the efficiency of using a miRNA with the specific intention of degrading a target mRNA, and for comparison of general co-expression trends to specific experimental observations.

## Availability

mirCoX, a web application described in this article, is available at http://wrenlab.org/mirCoX

## Competing interests

The authors declare that they have no competing interests.

## Authors' contributions

CG generated the database of correlations and targets and performed the experiments, RG implemented the web interface, CG, RG, MD and JW participated in study design and helped write the manuscript.

## Supplementary Material

Additional File 1 **SRA accessions used to generate correlations**. The SRAmetadb was searched for appropriate sequencing runs (see Methods) and mapped to the UCSC hg19 reference genome. This file contains 141 run accessions after filtering out runs with fewer than 60% mapped reads or fewer than 10M mapped reads. Click here for file

## Declarations

Publication of this work has been supported in part by the National Institutes of Health (NIH, grants # 1P20GM103636 and 8P20GM103456 to JDW) and by the Indian Council of Medical Research's Viral Disease Network Program (VIR/8/2011-ECD-1 to RGD).

This article has been published as part of *BMC Bioinformatics *Volume 14 Supplement 14, 2013: Proceedings of the Tenth Annual MCBIOS Conference. Discovery in a sea of data. The full contents of the supplement are available online at http://www.biomedcentral.com/bmcbioinformatics/supplements/14/S14.
